# Prediction of lymph node metastasis in operable cervical cancer using clinical parameters and deep learning with MRI data: a multicentre study

**DOI:** 10.1186/s13244-024-01618-7

**Published:** 2024-02-27

**Authors:** Fengying Qin, Xinyan Sun, Mingke Tian, Shan Jin, Jian Yu, Jing Song, Feng Wen, Hongming Xu, Tao Yu, Yue Dong

**Affiliations:** 1 Department of Radiology, Cancer Hospital of Dalian University of Technology (Liaoning Cancer Hospital & Institute), Shenyang, Liaoning 110042 China; 2 School of Biomedical Engineering, Faculty of Electronic Information and Electrical Engineering, Dalian University of Technology, Dalian, 116081 China; 3 Department of Radiology, Huludao Center Hospital, Huludao, 125001 China; 4 Department of Radiology, Shengjing Hospital of China Medical University, Shenyang, 110801 China

**Keywords:** Lymph node metastasis, Cervical cancer, Deep learning, Magnetic resonance imaging

## Abstract

**Objectives:**

To develop and validate a magnetic resonance imaging-based (MRI) deep multiple instance learning (D-MIL) model and combine it with clinical parameters for preoperative prediction of lymph node metastasis (LNM) in operable cervical cancer.

**Methods:**

A total of 392 patients with cervical cancer were retrospectively enrolled. Clinical parameters were analysed by logistical regression to construct a clinical model (M1). A ResNet50 structure is applied to extract features at the instance level without using manual annotations about the tumour region and then construct a D-MIL model (M2). A hybrid model (M3) was constructed by M1 and M2 scores. The diagnostic performance of each model was evaluated by the area under the receiver operating characteristic curve (AUC) and compared using the Delong method. Disease-free survival (DFS) was evaluated by the Kaplan‒Meier method.

**Results:**

SCC-Ag, maximum lymph node short diameter (LN_max_), and tumour volume were found to be independent predictors of M1 model. For the diagnosis of LNM, the AUC of the training/internal/external cohort of M1 was 0.736/0.690/0.732, the AUC of the training/internal/external cohort of M2 was 0.757/0.714/0.765, and the AUC of the training/internal/external cohort of M3 was 0.838/0.764/0.835. M3 showed better performance than M1 and M2. Through the survival analysis, patients with higher hybrid model scores had a shorter time to reach DFS.

**Conclusion:**

The proposed hybrid model could be used as a personalised non-invasive tool, which is helpful for predicting LNM in operable cervical cancer. The score of the hybrid model could also reflect the DFS of operable cervical cancer.

**Critical relevance statement:**

Lymph node metastasis is an important factor affecting the prognosis of cervical cancer. Preoperative prediction of lymph node status is helpful to make treatment decisions, improve prognosis, and prolong survival time.

**Key points:**

• The MRI-based deep-learning model can predict the LNM in operable cervical cancer.

• The hybrid model has the highest diagnostic efficiency for the LNM prediction.

• The score of the hybrid model can reflect the DFS of operable cervical cancer.

**Graphical Abstract:**

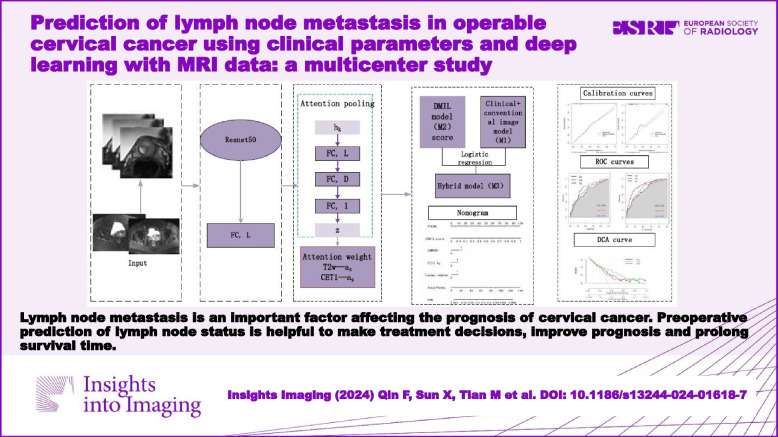

## Introduction

Cervical cancer is the fourth most common female malignancy worldwide [1, 2]. Early-stage cervical cancer is treated with radical hysterectomy (RH), but patients with lymph node metastasis (LNM) on histopathology after RH require adjuvant therapy [3]. However, RH combined with adjuvant therapy might lead to more serious comorbidities, such as genitourinary complications [4]. Because LNM is an important factor affecting prognosis, FIGO staging was revised in 2018, and patients with LNM were classified as stage IIIC and required concurrent chemoradiotherapy (CCRT) [5, 6]. Therefore, accurate diagnosis of LNM is crucial for improving prognosis and reducing mortality.

Magnetic resonance imaging (MRI) is an important imaging modality for the detection of LNM [7]. However, the diagnosis of LNM was mainly based on morphological indices such as size and shape, and the diagnostic effect was not satisfactory due to low sensitivity [8, 9]. Some studies have also used functional MRI parameters, such as the apparent diffusion coefficient (ADC) and K^trans^ value, to evaluate LNM in cervical cancer but are not suitable for wide application due to the long scanning time of functional sequences and the need for manual delineation of regions of interest (ROIs) [10–12]. F-18 fluorodeoxyglucose positron emission tomography/computed tomography (F-18 FDG PET/CT) has been shown to be more sensitive than CT or magnetic resonance imaging for the detection of lymph node metastasis in patients with cervical cancer; however, it has not been widely used in the preoperative assessment of cervical cancer due to the problems of high-dose radiation and high price [13].

The existing studies on the automatic diagnosis of LNM for cervical cancer mainly included radiomics feature-based approaches and deep learning (DL) models [14, 15]. However, handcrafted radiomic methods require time-consuming tumour boundary delineation and only detect generalised features, which cannot provide proper reproducible and repeatable features [16, 17]. A study developed an end-to-end DL model for diagnosing LNM in cervical cancer based on both intratumoural and peritumoural regions on contrast-enhanced T1-weighted (T1W) imaging [14]. Liu et al. established a CT-based DL model based on handcrafted 2D ROIs of the largest tumour area slice to predict LNM of cervical cancer, and the results showed that the performance of the DL model surpassed the diagnosis of experienced gynaecologists [18]. The above studies still used labelled images as the object of DL. We made a preliminary attempt to develop a deep multiple instance learning (D-MIL) model based on unlabelled MRI for predicting LNM in cervical cancer in a single-centre study, in which the AUC value reached 69% [19].

The main purpose of this study was to develop and validate an MRI-based D-MIL model and a hybrid model combining the D-MIL score with clinical data for the preoperative prediction of LNM in operable cervical cancer with multicentre data, and the secondary purpose was to assess the prognostic ability of the hybrid model scores regarding the disease-free survival (DFS) of cervical cancer.

## Materials and methods

### Research subjects

This retrospective study was performed after approval by the institutional review boards, and informed consent was waived. Data from patients who were diagnosed with cervical cancer were collected from December 2014 and June 2021 in centres 1, 2, and 3.

The following are the inclusion criteria: (1) pathologically diagnosed as cervical cancer, (2) underwent contrast-enhanced MR including T2-weighted (T2W) and T1-weighted (T1C) imaging before treatment, (3) no antineoplastic therapy before pelvic MR examination, and (4) patients who underwent RH with pelvic LN dissection ± para-aortic LN dissection ± postoperative adjuvant treatment.

The following are the exclusion criteria: (1) pathological types other than adenocarcinoma, squamous cell carcinoma, and adeno-squamous carcinoma; (2) MR images were not assessed due to poor image quality, such as motion artefacts, insufficient contrast, or noise; (3) clinical and pathologic data were not obtained from medical records; and (4) patients had other concurrent malignant tumours.

Patients were followed up with MRI or positron emission tomography and computed tomography every 3 to 4 months for the first 2 years, every 6 months from the third to fifth years, and then annually after surgery. DFS was defined as the time between the surgery and the first local–regional recurrence, distant metastasis, all-cause death, or the most recent follow-up utilised for censoring.

### Clinical data collection

Patient clinical and tumour characteristics were collected, including age, menopausal status, histologic type of the tumour, degree of differentiation, squamous cell carcinoma antigen (SCC-Ag), tumour size, tumour volume, and short diameter of maximum lymph node (LN_max_).

### MRI examination techniques

MRI scans were performed using a 3.0-T unit MRI scanner (Magnetom Trio, Siemens Medical Solutions, Germany) with an 8-channel phased array body coil and respiratory gating technology. Precontrast MRI scans included axial fast spin echo (FSE) T1-weighted (T1W) images, axial fat suppression fast spin echo T2-weighted (T2W) images, and sagittal FSE T2W images. Then, fat-suppressed contrast-enhanced T1W (CE-T1W) axial and sagittal images were obtained after approximately 20 mL of gadodiamide was injected via a pressure injector at a dosage of 2 mL/s followed by a 20-mL saline solution flush. A summary of the MRI parameters is presented in Table 1.

**Table 1:** MR imaging parameters

Sequence	Imaging plane	TR (ms)/TE (ms)	Section thickness (mm)	Gap (mm)	Field of view (mm)
Centeral1					
FS FSE T2-weighted	Axial	3000/106	5	2	294 × 448
Contrast-enhanced T1-weighted spin echo images	Axial	677/11	5	2	192 × 192
Central 2					
FS FSE T2-weighted	Axial	3120/72	5	2	280 × 440
Contrast-enhanced T1-weighted spin echo images	Axial	514/11	5	2	512 × 640
Central 3					
FS FSE T2-weighted	Axial	4326/102	5	1	288 × 254
Contrast-enhanced T1-weighted spin echo images	Axial	3.4/1.32	5	1.5	200 × 171

All imaging parameters were measured by a radiologist with 3 years of MR experience under the supervision of a senior radiologist with 20 years of experience who was blinded to the clinical and pathological data. The short diameter of LN_max_ and the maximum diameter of the tumour were measured on T2WI images in three directions. Tumour volume was calculated (multiplying the sum of tumour area measurement by the height including section thickness and the *z* gap between slices).

### Building the deep learning signature

Our team developed an attention-based MIL model to diagnose LNM in cervical cancer. The proposed MIL model adopts Residual Neural Network 50 (ResNet50) to extract features from T2W and CE-T1W images and attention-based pooling to make patient-level LNM predictions.

**Fig. 1:**
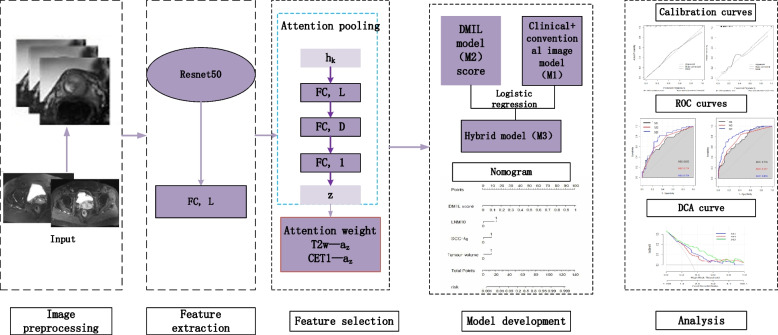
Illustration of the D-MIL model and the hybrid model

An overview of the MIL model network is shown in Fig. 1. Before starting the analysis, the image pixel values of all patients were normalised to maintain the consistency of pixel distributions across the different hospitals. A ResNet50 structure is applied to extract features at the slice level (instance level) without using manual annotations about the tumour region. For each patient, 2D MRI slices are converted into features and then fed into the MIL module. Attention values for features of MRI slices are computed. Then, the patient-level feature representation is aggregated according to the attentional pooling. For detailed information, please refer to the previous publication [19].

To better evaluate classification performance, we conduct fivefold cross-validation in the training cohort [10]. The model is trained using the Adam optimiser with a learning rate of 2 × 10^-5^, *β*1 = 0.9, *β*2 = 0.999, and weight decay of 1 × 10^-4^. The batch size is set as 1. The number of epochs is set as 200 with early stopping if the validation loss does not decrease after 30 epochs. Then, verification was carried out in the internal validation cohort and the external validation cohort.

### Statistical analysis

The SPSS 26.0 statistical software and R (4.2.2) were used for statistical analysis. The proposed RA-MIL model is implemented in Python with PyTorch (version 1.7.1). A Shapiro‒Wilk normality test was performed on continuous variables to examine the normal distribution of each variable. The cut-off values of SCC-Ag, maximum tumour diameter and tumour volume in this study through ROC analysis, and the point with the largest Jordan index was selected as the best truncation value cut-off point. The cut-off values of maximum tumour diameter were determined according to the cut-off value of the tumour size in the FIGO stage of cervical cancer, and the cut-off value of maximum LN short diameter was determined according to the accepted criteria of ≥ 10 mm as positive lymph nodes in the literature. Differences in clinical metrics between the two groups were compared using the chi-square test and corrected chi-square test. Logistic regression analysis was used for multivariate analysis. The forwards LR method was used to screen independent risk factors, and a clinical predictive model was established. The established clinical model (M1) and D-MIL model score (M2) were combined to establish a hybrid model (M3). Finally, the hybrid model was visualised by a nomogram.

To assess the performance of each model, ROC curve analysis was used. The AUC, sensitivity, and specificity were all calculated. The Delong test was used to compare the AUCs of the prediction models [20]. To test the compatibility of the anticipated findings with the actual data, the “rms” program was used to create a nomogram and draw calibration curves. Decision curve analysis (DCA) with net benefits for threshold probabilities was used to determine the clinical usefulness of the clinical model, D-MIL model, and hybrid model (Fig. 1). DFS was estimated using the Kaplan‒Meier technique and log-rank testing. *p* < 0.05 indicated a statistically significant difference.

## Results

### Patient cohort and baseline characteristics

**Fig. 2:**
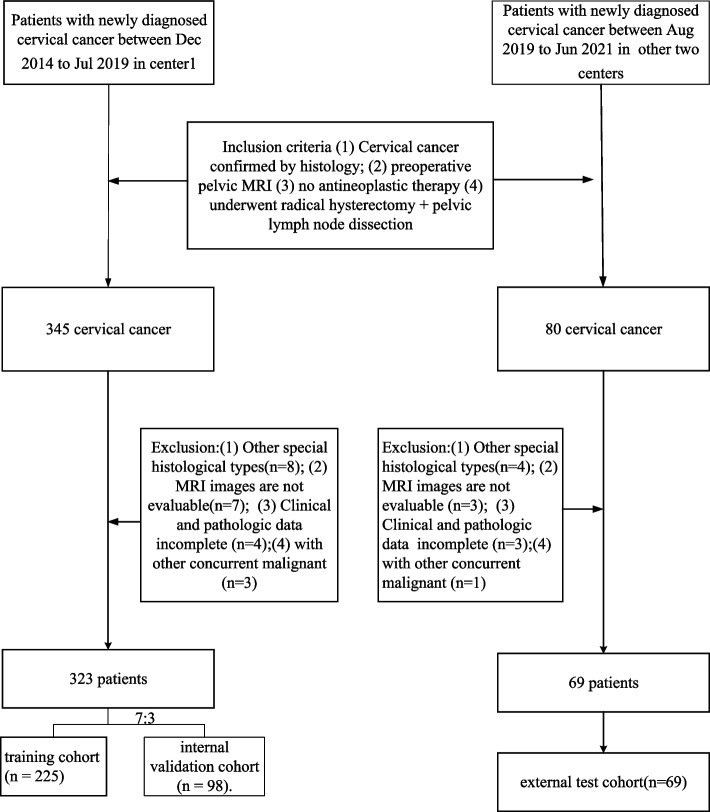
The workflow diagram of patient recruitment

Of the 425 patients who underwent preoperative MRI, 12 cases were excluded for special pathological types, 10 cases for poor image quality, 7 cases for incomplete clinical or pathological data, and 4 cases for concurrent other tumours. Finally, a total of 392 patients (mean age 51.6 ± 9.6 years, range 25–75 years) were included in the study. Squamous cell carcinoma, adenocarcinoma, and adeno-squamous carcinoma were identified in 345, 45, and 2 cases, respectively. A total of 323 patients in centre 1 were randomly resampled and divided into a training cohort (*n* = 225) and an internal test cohort (*n* = 98) at a ratio of 7:3. Following the same eligibility criteria, 69 patients in the other 2 centres were collected to constitute an external test cohort (*n* = 69). Patient recruitment is shown in Fig. 2.

### Clinical characteristics and clinical model building

The clinical data of the patients are presented in Table 2.

**Table 2:** Clinical characteristics of the training and internal validation cohorts and external validation cohort in other centres

Parameters	Training cohort (*n* = 225)			Internal validation cohort (*n* = 98)			external validation cohort (*n* = 69)		
	LNM group (*n* = 75)	Non-LNM group (*n* = 150)	*p*	LNM group (*n* = 33)	Non-LNM group (*n* = 65)	*p*	LNM group (*n* = 20)	Non-LNM group (*n* = 49)	*p*
Age (years)			0.920			0.72			0.140
≥ 50	48	94		17	36		12	38	
< 50	27	56		16	29		8	11	
Menstrual state			0.700			0.73			0.770
Menopause	49	48		19	35		13	37	
Premenopausal	26	102		14	30		7	12	
SCC-Ag (ng/L)			< 0.001			0.02			0.020
≥ 1.5	48	47		18	20		13	17	
< 1.5	27	103		15	45		7	32	
Pathological type			0.460			0.22			0.880
Squamous cell carcinoma	68	131		30	53		18	44	
Adenocarcinoma	7	19		3	12		2	5	
Histologic grade			0.510			0.16			0.400
High/middle	40	87		26	58		14	39	
Low	35	63		7	7		6	10	
LN_max_ short diameter (mm)			< 0.001			0.008			0.004
≥ 10	18	5		23	6		8	5	
< 10	57	145		10	59		12	44	
Maximum tumour diameter (mm)			< 0.001			0.030			0.052
≥ 40	38	26		14	14		7	7	
< 40	37	124		19	51		13	42	
Tumour volume (cm^3^)			< 0.001			0.005			0.020
≥ 15	31	35		13	20		10	11	
< 15	44	115		20	45		10	38	

In the training, internal, and external cohorts, there were significant differences in SCC-Ag ≥ 1.5 ng/L, maximum tumour diameter ≥ 40 mm, tumour volume > 15 cm^3^, and maximum LN short diameter ≥ 10 mm between the LNM group and the non-LNM group. The parameters with significant differences in the training group were included in multivariate logistic regression analysis to establish a clinical model (M1 =  -1.777 + 1.031 × tumour volume ≥ 15 cm^3^ + 1.695 × LNM short diameter ≥ 10 mm). The area under the AUCs of M1 reached 0.757, 0.714, and 0.765 in the training, internal validation, and external test cohorts, respectively.

### D-MIL model and hybrid model construction

**Fig. 3:**
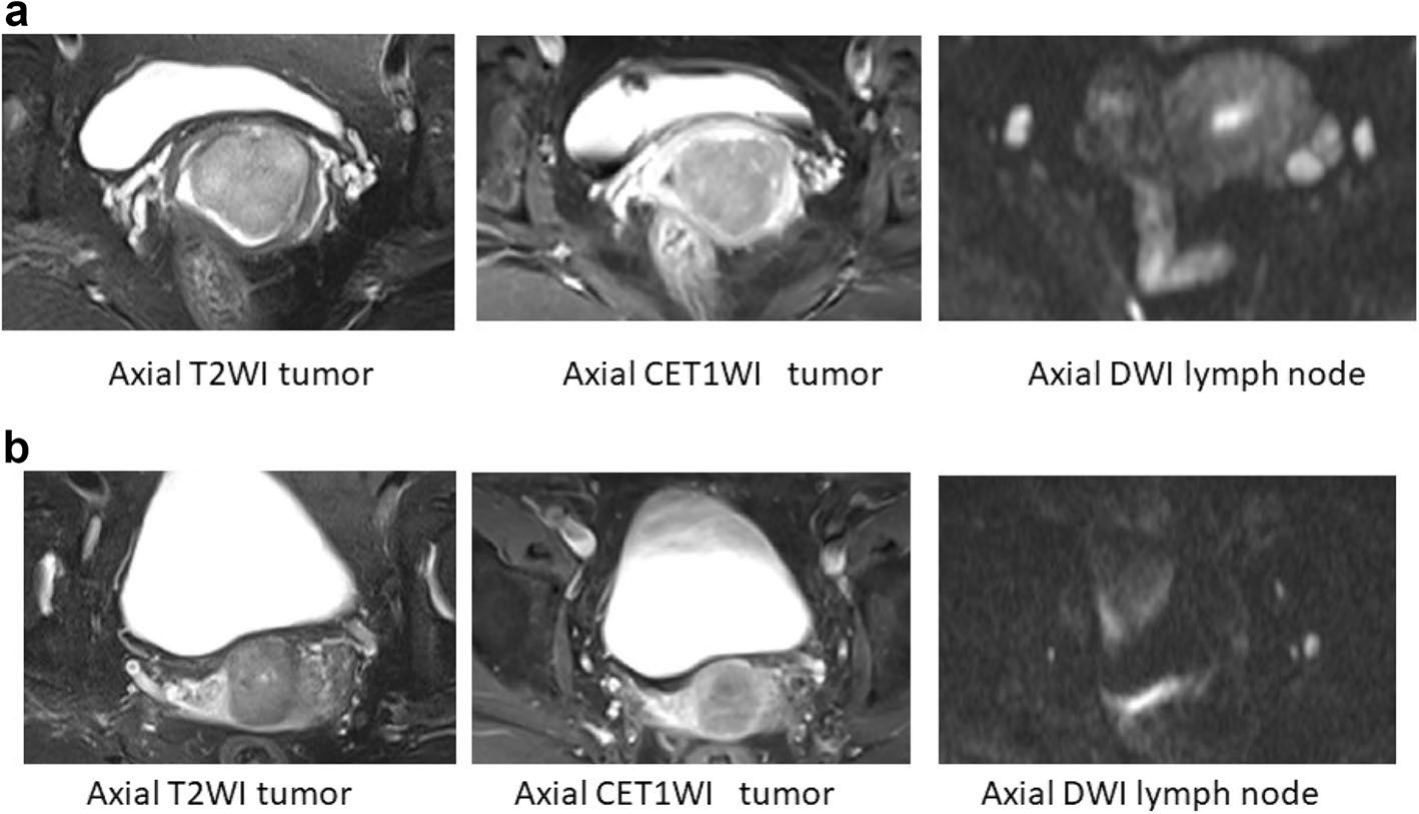
**a** Age, 64 years; stage, IIA2; tumour volume, 15.76 cm^3^; SCC-Ag, 2.9 ng/L; MRI-LN, 10 mm, 9 mm, true-positive; D-MIL score of T2WI tumour, 0.691, true-positive; hybrid model score, 0.778, true-positive. **b** Age, 53 years; stage, IIA2; tumour volume, 13.11cm^3^; SCC-Ag, 0.6 ng/L; MRI-LN, 8 mm, true- negative; D-MIL score of T2WI tumour, 0.202, true-negative; hybrid model score, 0.161, true-negative

The AUCs of the D-MIL model (M2) in the training cohort, internal validation cohort, and external test cohort from other institutions were 0.736, 0.690, and 0.732, respectively. Finally, the clinical model and the D-MIL model score were combined to establish a hybrid model by logistic regression. The AUCs of M3 in the training, internal validation, and external test cohorts were 0.838, 0.764, and 0.835, respectively. Figure 3 shows the MR images of two patients who had similar clinicopathologic characteristics, making it difficult to identify LN status by clinical characteristics and visual observation on MRI. However, the D-MIL and hybrid models were able to generate discriminative predictive values (Fig. 3A, B).

### Comparison of model performance

**Fig. 4:**
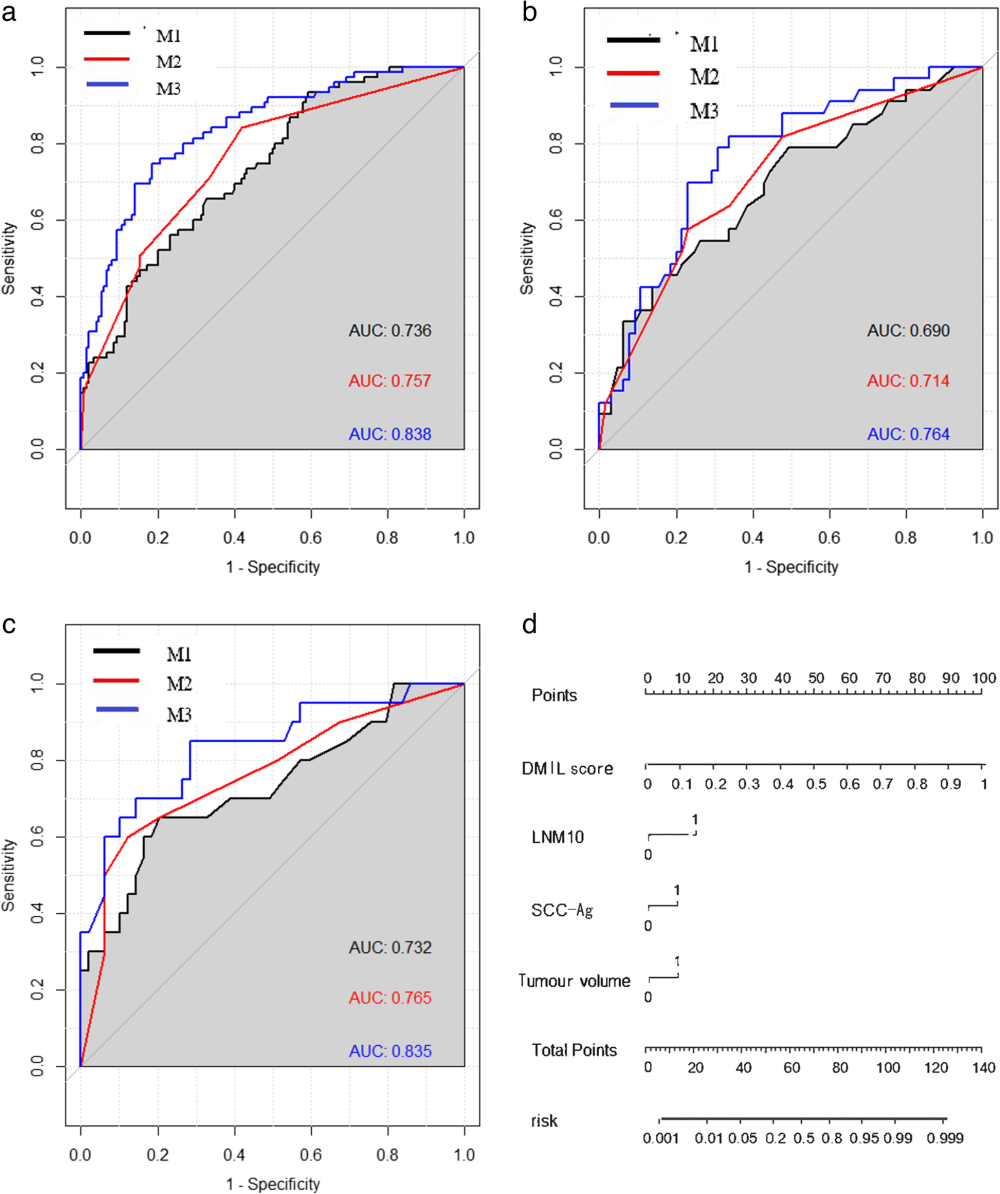
**a** ROC curves of training cohorts. **b** ROC curves of internal validation cohorts. **c** ROC curves of external validation cohorts. **d** Nomogram for LNM of patients with cervical cancer with LNM (model 3)

**Fig. 5:**
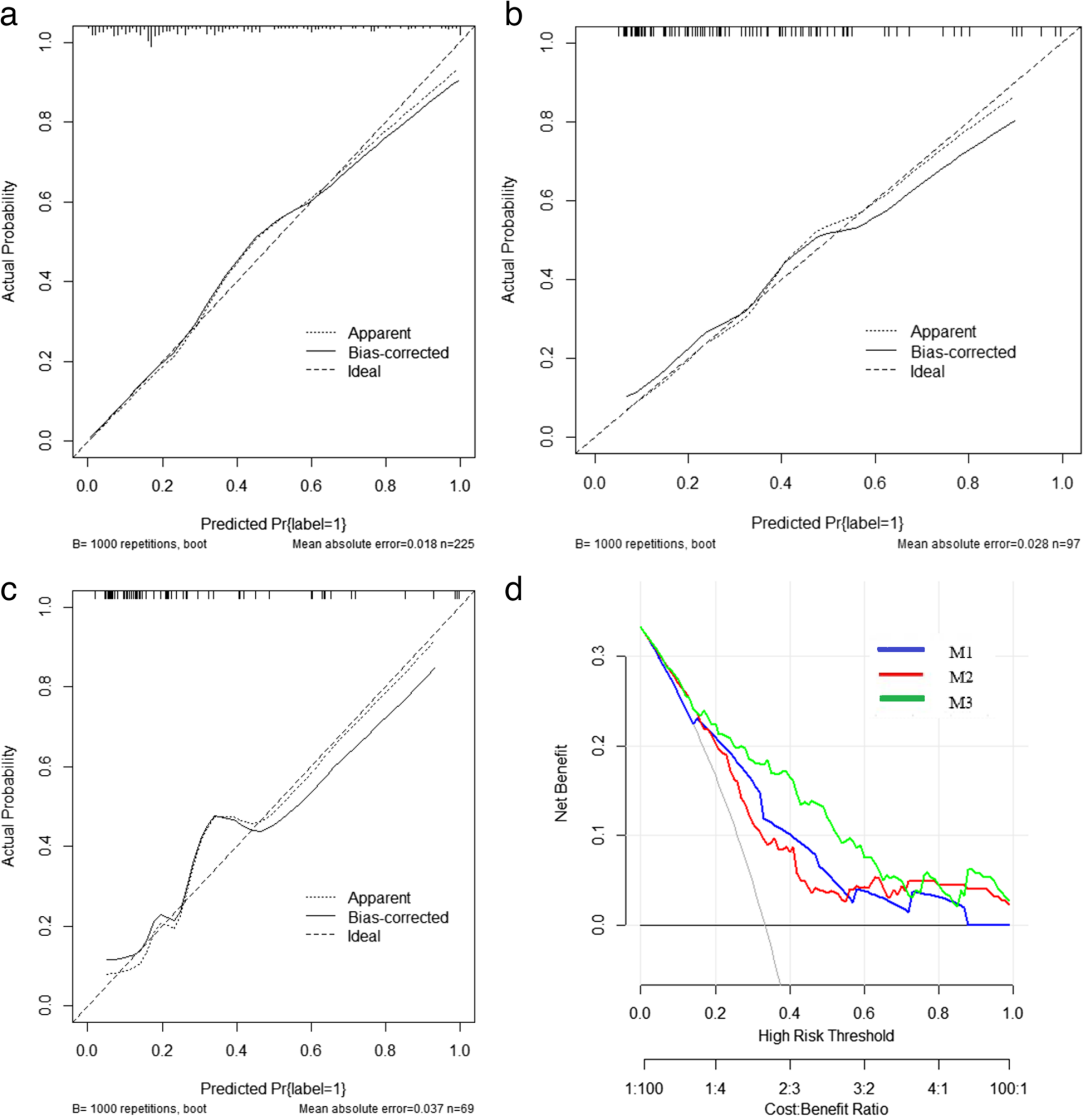
**a** Calibration curves of the nomogram in the training cohorts. **b** Calibration curves of the nomogram in the internal validation cohorts. **c** Calibration curves of the nomogram in the internal validation cohorts. **d** Decision curve analysis

As shown in Table 3 and Fig. 4, M3 had the highest diagnostic efficiency. According to the Delong test, there were significant differences between the clinical model and the hybrid model (training cohort, *p* < 0.001; internal validation cohort, *p* = 0.007; external validation cohort, *p* = 0.016). At the same time, the results proved that M3 can improve the diagnostic efficiency of the simple D-MIL model (M2) to a certain extent (training cohort, *p* < 0.001; external validation cohort, *p* = 0.04). The decision curves showed that the patients could benefit more from M3 than both M1 and M2 (Fig. 5D). As shown in Fig. 5A–C, the calibration curves demonstrated that M3 had good consistency with the gold standard of LNM.

**Table 3:** Diagnostic efficacy of clinical model, DL model, and hybrid model

Model	Training cohort (*n* = 225)				Internal validation cohort (*n* = 98)				External validation cohort (*n* = 69)			
	Sensitivity	Specificity	AUC	*p*	Sensitivity	Specificity	AUC	*p*	Sensitivity	Specificity	AUC	*p*
M1	0.580	0.840	0.736 (0.736–0.838)		0.727	0.646	0.690 (0.609–0.820)		0.600	0.870	0.732 (0.567–0.839)	
M2	0.692	0.818	0.757 (0.669–0.802)		0.693	0.821	0.714 (0.576–0.803)		0.619	0.816	0.765 (0.591–0.873)	
M3	0.747	0.813	0.838 (0.783–0.893)		0.750	0.800	0.764 (0.665–0.862)		0.85	0.730	0.835 (0.721–0.948)	
M1 vs. M2				0.650				0.750				0.650
M2 vs. M3				< 0.001				0.280				0.040
M1 vs. M3				< 0.001								0.016

### Prognostic value of the hybrid model

**Fig. 6:**
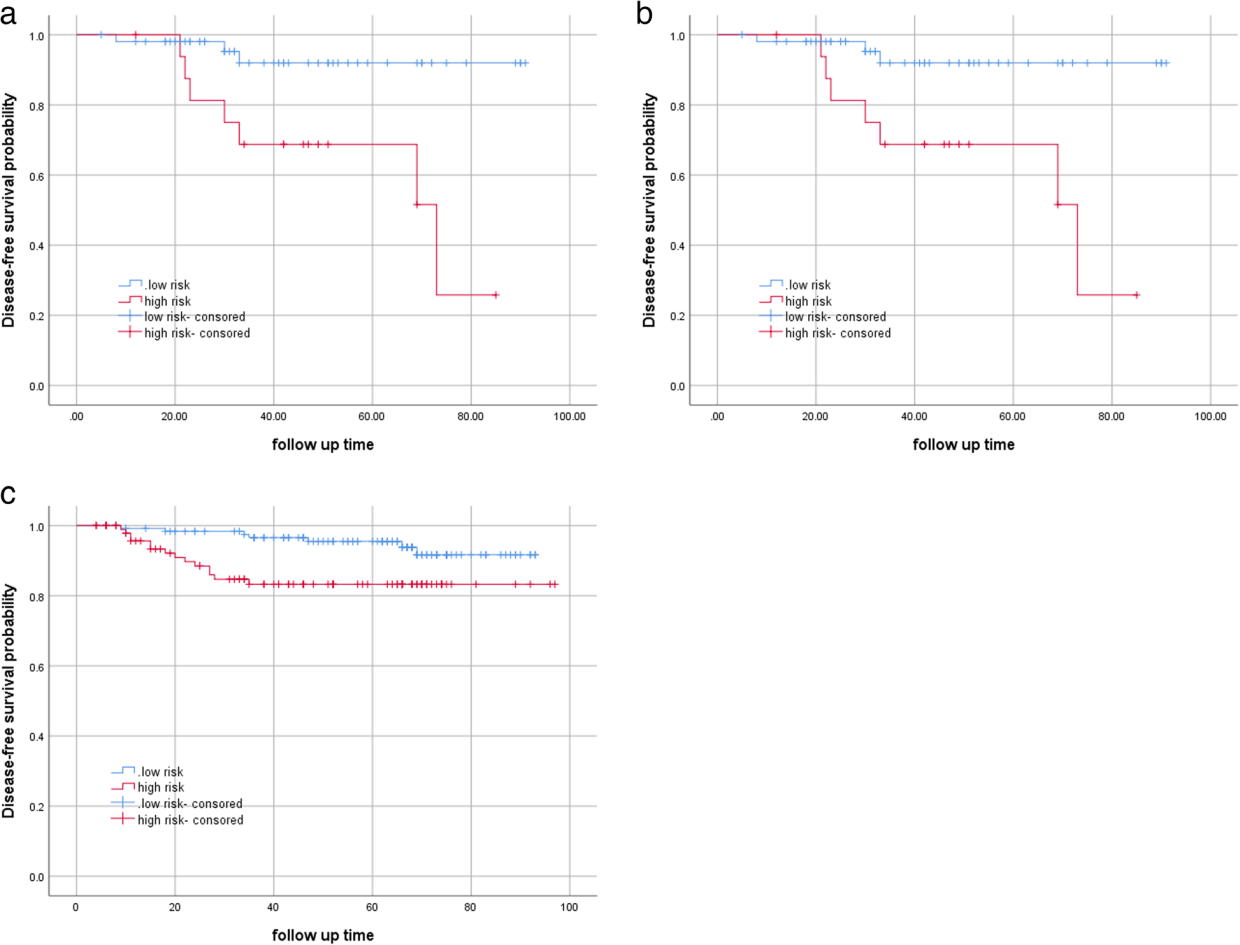
**a** K-M curves for the hybrid model in the training cohort. **b** K-M curves for the hybrid model in the internal test cohort. **c** K-M curves for the hybrid model in the external test cohort

As of December 2022, the median follow-up time was 49 (range 8–65) months in the training cohort, 49 (range 8–65) months in the internal cohort, and 43 (range 12–63) months in the external validation cohort. LN status of cervical cancer has been reported to be a crucial prognostic factor, we performed survival analyses to assess the prognostic ability of the hybrid model regarding 3-year DFS. The median score was used to stratify patients into low- and high-risk groups. Figure 6A–C shows a significant difference between low- and high-risk patients from the hybrid model in the training cohort (hazard ratio, 3.14; 95% CI, 1.26–7.78; *p* = 0.014), internal validation cohort (hazard ratio, 7.25; 95% CI, 1.50–34.95; *p* = 0.014), and external validation cohort (hazard ratio, 6.32; 95% CI, 1.63–23.46; *p* = 0.008). Patients with higher scores had a shorter time to reach DFS.

## Discussion

The study developed a clinical model predicting LNM with SCC-Ag, tumour volume, and short diameter of LN_max_. SCC-Ag is the most important tumour marker of cervical cancer and is highly correlated with the severity of the disease, and its elevation indicates a poor prognosis [21]. Ran et al. analysed the clinical data of 200 patients with cervical cancer and found that SCC-Ag- and MRI-reported LN status were independent influencing factors for LNM [22]. Consistent with previous studies, our study also found that SCC-Ag 1.5 ng/L was an independent factor impacting LNM. Large tumour size was also a risk factor for LNM, with tumour diameters greater than 4 cm considerably increasing the incidence of pelvic LNM [23]. These findings are consistent with our study’s conclusion that a larger tumour diameter is related to a higher probability of LNM. However, multivariate analysis showed that the maximum diameter of the tumour was not an independent factor affecting LN status, which may be because the tumour volume can better reflect the biological behaviour of the tumour. The larger the tumour volume is, the deeper the surrounding invasion and the more LNM and lymphovascular space invasion (LVSI), and tumour volume can better predict LVSI and LNM status than the largest tumour diameter [24]. LNs with a short diameter ≥ 10 mm are generally regarded as LNM [25], so we divided the maximum short diameter of lymph nodes on MR images into two groups according to the cut-off value of 10 mm. The results showed that the probability of LNM in patients with an LN short diameter ≥ 10 mm was significantly higher than that in patients with an LN short diameter < 10 mm. However, judging lymph node metastasis only by shape and size will lead to some false negatives [26]. Eighty-nine patients in our cohort showed that the LN_max_ was < 10 mm, but postoperative pathology confirmed that the lymph nodes were positive.

This study combined ResNet50 with an attention mechanism to develop an end-to-end deep multi-instance learning model (M2) for the preoperative prediction of LNM in cervical cancer. M2 achieved a satisfactory evaluation of classification performance with 0.693 sensitivity and 0.821 specificity in the internal validation cohort and 0.619 sensitivity and 0.816 specificity in the external validation cohort.

Previous studies mainly used radiomic analysis to predict LNM of cervical cancer based on CT or MR images, which achieved good differentiation [27, 28]. Wang et al. proposed a non-invasive hybrid model based on the clinicopathologic factors and radiomics signature for preoperatively predicting LNM in cervical cancer, which showed a significant improvement over the model based only on clincopathological factors in the training cohort (AUC, 0.893 vs. 0.616) and validation cohort (AUC, 0.922 vs. 0.799) [27]. Another study developed and validated a radiomics-based nomogram incorporating the multiparametric MRI radiomics signature and the MRI-reported LN status for prediction of LNM in cervical cancer, which showed good calibration and discrimination in both training and validation sets, with AUCs of 0.865 and 0.861 [28]. The above radiomic models could only reflect lower-rank image features, and the overall model performance depends on the processing quality of several sequential steps including tumour segmentation, feature extraction, feature selection, and model establishment. Compared with the radiomic model, deep learning reduces the subjectivity and time of manual feature selection and has a hierarchical structure of non-linear features, which is helpful for better modelling of very complex data patterns [29]. However, the manual drawing of regions of interest was still used in some similar deep learning studies, which was both time-consuming and subject to inter- and intra-observer variability [15, 29]. For this reason, the paper intends to explore a D-MIL method with strong and robust feature learning ability from unlabelled MRI data, which can automatically learn tumour features related to LN status and avoid complex tumour boundary segmentation. Additionally, our model also integrates the slices of the upper and lower layers near the tumour into the deep learning network to avoid the loss of important information caused by only including the region of the tumour or even the 2D image of the largest level of the tumour. In addition to information about the tumour itself, a previous study demonstrated that peritumoural information can also reflect the biological behaviour of cervical cancer, and the deep learning model that used both intratumoural and peritumoural regions on CE-T1 images showed the best performance for the prediction of LNM among three image sequences (CET1W, T2W, and DWI) [15].

Our team developed an attention-based MIL model to diagnose LNM in cervical cancer. The proposed MIL model adopts Residual Neural Network 50 (ResNet50) to extract features from T2W and CE-T1W images and attention-based pooling to make patient-level LNM predictions. The MIL framework is widely used in histopathologic whole-slide images [30]. Some literatures have applied the MIL method to recognise survival-relevant high-risk subregion in brain glioblastoma, because there is a certain heterogeneity in the tumour; cervical cancer has also been proved to be an internal heterogeneous tumour, so our team added MIL when designing the prediction model [31, 32]. In this study, the MRI of a patient’s tumour is treated as a bag, with each slice of the lesion considered an instance. Through MIL module preprocessing, each slice was given a different weight based on its importance. Then, the modality attention module assigns higher weights to the MRI modality with the highest predictive effect. The features of the instances are then aggregated to form the bag features, which are used to produce the final prediction results. The DL network with the MIL ideology and attention mechanism enables LNM diagnosis at the patient level and identifies target positive slices for better interpretability. Both positive slices and significant regions can be automatically captured to predict LNM status. Our designed model provides a non-invasive tool for preoperative LNM diagnosis of cervical cancer, which also has the potential to be transferred into other classification tasks in disease diagnosis.

It is worth noting that model 1 based on clinical factors and conventional MRI indicators and model 2 based on deep learning features had similar diagnostic efficacy in our cohorts, which means that a simple deep learning model could not significantly improve the diagnostic performance of LNM. The hybrid model (M3) from the M1 and M2 scores showed encouraging predictive performance in diagnosing LNM, reaching AUCs of 0.838 and 0.764 in the training and internal validation cohorts and 0.835 in the external test cohort. M3 includes clinical key information, the subjective experience of radiologists and high-dimensional features from deep learning that cannot be identified visually. Previous studies have also shown that a nomogram established by combining clinical parameters and deep learning model based on MR images can be used as an individual tool for the non-invasive prediction of LNM in cervical cancer, which is helpful for making treatment plans [18, 33].

LNM is one of the main causes of poor prognosis in patients with cervical cancer [34, 35]. In the early, middle, and advanced stages, the 5-year survival rate of cervical cancer without lymph node metastasis is close to 90%, while the 5-year survival rate drops sharply to 65% in cervical cancer with lymph node metastasis [34]. Our results found that the score of the hybrid model was significantly related to the 3-year DFS of cervical cancer, and the patients with higher scores have lower DFS, indicating that the hybrid model was a good prognostic indicator. Tian et al. developed a hybrid model combining the CE-T1W tumour + peritumoural model with MRI-LN status that was related to the survival outcome of early-stage cervical cancer [15]. According to the hybrid model established in this study, LNM can be predicted by MRI image data and clinical indicators before treatment. Patients with LNMs can be treated with CCRT to avoid the double side effects of surgery plus postoperative radiotherapy and chemotherapy.

Several limitations of this study should be considered. First, the sample size of the study is still small, which might create potential bias. Second, this study is a retrospective study and requires prospective multicentre studies with a large cohort to further confirm our deep learning model. Third, PET-CT is sensitive to lymph node metastasis of cervical cancer, but it is not used for preoperative evaluation on a large scale because of its high cost and radiation. It is expected that PET-CT can be used to evaluate distant lymph node metastasis before surgery in future studies.

Last, due to the lack of functional MRI data, such as DWI images, multimodal data may have better results.

## Conclusion

This study used unlabelled MRI data to propose an attention-based D-MIL model for LNM prediction in operable cervical cancer. The hybrid model can further improve the ability to predict LNM in cervical cancer. At the same time, the hybrid model scores could also reflect the DFS of operable cervical cancer.

## Data Availability

The data and/or analysed during the current study are available from the corresponding author upon reasonable request.

## Abbreviations

ADC: Apparent diffusivity coefficient
AUC: Area under the curve
CCRT: Concurrent chemoradiotherapy
CET1W: Contrast-enhanced T1-weighted imaging
D-MIL: Model deep multiple instance learning
DCA: Decision curve analysis
DFS: Disease-free survival
DL: Deep learning
FSE: Fast spin echo
LN: Lymph node
LNM: Lymph node metastasis
LVSI: Lymphovascular space invasion
MIL: Multiple instance learning
MRI: Magnetic resonance imaging
ResNet50: Residual Neural Network 50
RH: Radical hysterectomy
ROC: Receiver operating characteristic
ROI: Regions of interest
SCC-Ag: Squamous cell carcinoma antigen
T1W: T1-weighted
T2W: T2-weighted
